# The prognostic impact of Akt isoforms, PI3K and PTEN related to female steroid hormone receptors in soft tissue sarcomas

**DOI:** 10.1186/1479-5876-9-200

**Published:** 2011-11-22

**Authors:** Andrej Valkov, Thomas K Kilvaer, Sveinung W Sorbye, Tom Donnem, Eivind Smeland, Roy M Bremnes, Lill-Tove Busund

**Affiliations:** 1 Dept of Clinical Pathology, University Hospital of Northern Norway, Tromsø, Norway; 2 Institute of Medical Biology, University of Tromsø, Norway; 3 Dept of Oncology, University Hospital of Northern Norway, Tromsø, Norway; 4 Institute of Clinical Medicine, University of Tromsø, Norway

**Keywords:** soft tissue sarcomas, Akt isoforms, PI3K, PTEN, ER, PgR, disease-specific survival

## Abstract

**Background:**

The PI3K/Akt pathway is involved in cellular survival pathways by inhibiting apoptotic processes and stimulating cell growth and proliferation. Its negative prognostic value has been proven in many types of cancer. In soft tissue sarcomas, the expression profiles of the PI3K/Akt pathway components are poorly defined and their significance uncertain. We aimed to investigate the prognostic impact of Akt (Akt1) phosphorylated at threonine^308 ^and serine^473^, Akt2, Akt3, PI3K and PTEN, alone and in coexpression with ER and PgR in non-gastrointestinal stromal tumor soft tissue sarcomas (non-GIST STSs).

**Patients and methods:**

Tumor samples and clinical data from 249 patients with non-GIST STS were obtained, and tissue microarrays (TMAs) were constructed. Immunohistochemistry (IHC) was used to evaluate marker expression in tumor cells.

**Results:**

In univariate analyses, the expression levels of p-Akt Thr^308 ^(P = 0.002), Akt2 (P = 0.008) and PI3K (P < 0.001) were significant prognostic factors. In the multivariate analysis, high PI3K expression was an independent negative prognosticator (HR = 1.5, 95% CI = 1.0-2.2, P = 0.042) in addition to advanced age, tumor depth, high malignancy grade, metastasis at diagnosis, surgery and positive resection margins. p-Akt Thr^308 ^expression had strong unfavorable effect in men only (P = 0.009). In contrast, p-Akt Ser^473 ^expression had strong unfavorable impact in women (P = 0.023). PgR-/p-Akt Ser^473^+ phenotype tended to have less favorable impact in women (P = 0.087), but was the most favorable one in men (P = 0.010).

**Conclusion:**

Expression of PI3K was significantly associated with aggressive behavior and shorter DSS in non-GIST STSs. The site of Akt phosphorylation seems to have gender-dependent impact on survival in STS patients.

## Background

Soft tissue sarcomas (STS) are malignant tumors arising from extraskeletal connective tissues. They are heterogeneous neoplasms, consisting of more than 50 subtypes, and comprise less than 1% of adult malignancies [1,2]. Approximately 50% of the STS patients will succumb to their disease because of metastasis or local progression [3]. The prognostic factors determining tumor evolution and ultimately patients' fate include tumor grade, size, location, depth, histological entity, positive resection margins and presence of local relapse [4-10]. In addition, an array of recurrent gene aberrations are found to be prognostic and predictive biomarkers in STSs [11-13].

Akt is a serine/threonine protein kinase that exists in three possible isoforms, including Akt1, Akt2, and Akt3. Akt can be activated by phosphorylation at threonine ^308 ^or at serine ^473 ^for Akt1 or homologous sites for Akt2 and Akt3 by phosphatases which along with Akt isoforms, belong to the phosphoinositide 3-kinase (PI3K)/Akt pathway. The PI3K/Akt pathway has been linked to an extraordinarily diverse group of cellular functions, including cell growth, proliferation, differentiation, motility, survival, intracellular trafficking and angiogenesis [14]. Both PI3K and Akt isoforms have been implicated as major players in many types of cancer [15-17].

The PI3K/Akt pathway seems to be more often deregulated in cancer than any other pathway [18]. However, in the literature there is disagreement regarding the prognostic impact of Akt expression. While the majority of studies agree that Akt expression overtly indicates a poor prognosis [19-21], there are several studies showing the opposite effect [22,23]. Expressions of PI3K/Akt pathway components have rarely been investigated in STSs and there are almost no studies devoted to their prognostic value [24].

Different physiological function of the Akt family kinases implies that the expression of its isoforms may also have different prognostic impact in cancer. The significance of this variation for the survival of the STS patients is not well investigated and it is not clear whether the site of phosphorylation and the pattern of expression can play prognostic roles.

In previous studies, we have shown the prognostic value of female steroid hormone receptors in STSs, both alone and in the coexpression with TGF-β and fascin [25,26]. Such prognostic impact is not surprising, since both ER and PgR regulate growth and cell differentiation upon ligand-dependent and ligand-independent activation and are in essence growth factors. In this context we wanted to explore the correlations between female hormone receptors and the members of PI3K/Akt signaling pathway. To our knowledge, these correlations have not been described previously.

In this study, we investigate the prognostic impact of all isoforms of Akt (phosphorylated at threonine ^308 ^and Akt phosphorylated at serine ^473^, non-phosphorylated Akt2, and total Akt3), PI3K, PTEN, ER and PgR in 249 non-GIST STS patients. GIST cases were excluded from the study since patients with this subtype of sarcoma receive a specific treatment regimen which resulted in significantly better survival.

## Materials and methods

### Patients and clinical samples

Primary tumor tissue from anonymized patients diagnosed with non-GIST STS at the University Hospital of North Norway (UNN) 1973-2006 and The Hospitals of Arkhangelsk region, Russia, were used in this retrospective study. In total, 496 patients were registered from the hospital databases. Of these, 247 patients were excluded due to missing clinical data (n = 86) or inadequate material for histological examination (n = 161). Thus, 249 STS patients with full clinical records and adequate paraffin-embedded tissue blocks were eligible.

This report includes follow-up data as of September 2009. The median follow-up was 38 (range 0.1 - 392) months. Formalin-fixed and paraffin-embedded tumor specimens were obtained from the archives of the Departments of Pathology at UNN and the Arkhangelsk hospitals. The tumors were graded according to the French Fèdèration Nationales des Centres de Lutte Contre le Cancer (FNCLCC)[27].

### Microarray construction

All sarcomas were histologically reviewed by two trained pathologists (S.S. and A.V.) and the most representative areas of viable tumor cells (neoplastic cells) were carefully selected and marked on the hematoxylin and eosin (H&E)-stained slides and sampled for the tissue microarray blocks (TMAs). The TMAs were assembled using a tissue-arraying instrument (Beecher Instruments, Silver Springs, MD). The Detailed methodology has been previously reported [28]. Briefly, we used a 0.6 mm diameter stylet, and the study specimens were routinely sampled with two replicate core samples (different areas) of neoplastic tissue. To include all core samples, 12 tissue array blocks were constructed. Multiple 4-μm sections were cut with a Micron microtome (HM355S) and stained using specific antibodies for immunohistochemistry (IHC) analyses.

### Immunohistochemistry (IHC)

The applied antibodies were subjected to in-house validation by the manufacturer for IHC analysis on paraffin-embedded material. The applied antibodies had been subjected to in-house validation by the manufacturer for IHC analysis on paraffin-embedded material. The antibodies used in the study were as follows: Phospho-Akt (Ser473) (1:5; Rabbit monoclonal, clone 736E11; #3787; Cell Signalling Technology, Danvers, U.S.A.), detects Akt 1 only when phosphorylated at serine 473, and Akt2 and Akt3 only when phosphorylated at equivalent sites. Phospho-Akt (Thr308) (1:50; Rabbit monoclonal, clone 244F9; #4056; Cell Signalling Technology), recognizes all three Akt isoforms when phosphorylated at this site. Akt2 (1:18; Rabbit monoclonal, clone 54G8; #4057; Cell Signalling Technology), preferentially binds to non-phosphorylated endogenous levels of Akt2. It does not cross-react with recombinant Akt1 or Akt3. Akt3 (1:8; Rabbit polyclonal, #4059; Cell Signalling Technology), detects endogenous levels of total Akt3, but does not recognize the truncated form of rat Akt3. The antibody does not cross-react with recombinant Akt1 or Akt2. PTEN (1:10, Rabbit monoclonal; #9559; Cell Signalling Technology), detects endogenous levels of total PTEN protein. PI3K (1:25; Rabbit polyclonal; #4254; Cell Signalling Technology), detects endogenous levels of total PI3K.

Sections were deparaffinised with xylene and rehydrated with ethanol. Antigen retrieval was performed by placing the specimen in 0.01mol/l citrate buffer at pH 6.0 and exposed to two repeated microwave heatings of 10 minutes at 450W. The DAKO EnVision + System-HRP (DAB) kit was used as endogen peroxidase blocking. Primary antibodies were incubated overnight at 4°C (except PI3K, for 32 minutes at room temperature). The DAKO EnVision+ System-HRP (DAB) kit was used to visualize the antigens for all stains. This yielded a brown reaction product at the site of the target antigen. As negative staining controls, the primary antibodies were replaced with the primary antibody diluent. Finally, all slides were counterstained with hematoxylin to visualize the nuclei. For each antibody, including negative controls, all TMA staining were performed in one single experiment. The immunohistochemical staining for ER and PgR was performed as described earlier[25].

### Scoring of IHC

**Figure 1:**
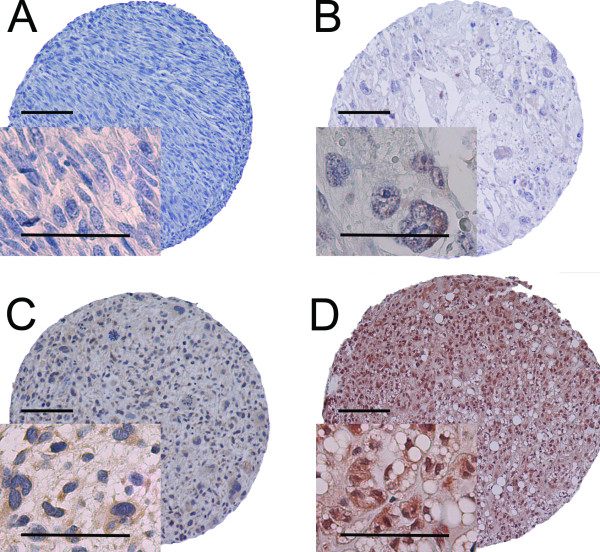
**IHC analysis of TMA of non-GIST STS representing different expressions of markers belonging to PI3K/Akt pathway in tumor cells**. A, Leiomyosarcoma, histological grade I, PTEN, negative staining, score 0; B, Pleomorphic liposarcoma, histological grade III, p-Akt Thr^308^, weak nuclear staining, score 1; C, Undifferentiated pleomorphic sarcoma, histological grade III, PI3K, moderate cytoplasmic staining; score 2; D, Dedifferentiated liposarcoma, histological grade II, p-Akt Ser^473^, strong both nuclear and cytoplasmic staining, score 3. All calibration bars correspond to 100 μm in the overview images (objective 10×) and 50 μm in the pictures taken at high magnification (objective 40×). Abbreviations: IHC, immunohistochemistry; TMA, tissue microarray; non-GIST STS, non gastro-intestinal stromal tumor soft-tissue sarcoma; PTEN, phosphatase and tensin homolog; p-Akt Thr^308^, Akt phosphorylated at threonin 308; PI3K, phosphoinositide 3-kinase; p-Akt Ser^473^, Akt phosphorylated at serin 473.

The ARIOL imaging system (Genetix, San Jose, CA) was used to scan the slides with immunohistochemically stained TMAs. The specimens were scanned at a low resolution (1.25×) and high resolution (20×) using Olympus BX 61 microscope with an automated platform (Prior). The slides were loaded in the automated slide loader (Applied Imaging SL 50). Representative and viable tissue sections were scored manually on computer screen, semiquantitatively for cytoplasmic staining for PI3K/Akt pathway components and for nuclear staining for ER and PgR. The dominant staining intensity in neoplastic cells was scored subjectively as: 0 = negative; 1 = weak; 2 = intermediate; 3 = strong (Figure 1). For ER and PgR, the modified All Red scoring system [25] was used. All samples were anonymized and independently scored by two pathologists (A.V. and S.S.). In cases where score difference was equal to or exceeding 2, the slides were re-examined and a consensus was reached by the observers. When assessing a score for a given core, the observers were blinded to the scores of the other variables and to outcome. Mean score for duplicate cores from each individual was calculated.

### Statistical methods

All statistical analyses were done using the statistical package SPSS (Chicago, IL), version 16. The IHC scores from each observer were compared for interobserver reliability by use of a two-way random effect model with absolute agreement definition. The intraclass correlation coefficient (reliability coefficient) was obtained from these results. The Chi-square test and Fishers Exact test were used to examine the association between molecular marker expression and various clinicopathological parameters. Univariate analyses were done by using the Kaplan-Meier method, and statistical significance between survival curves was assessed by the log rank test. Disease-specific survival (DSS) was determined from the date of histological confirmed STS diagnosis to the time of STS death. To assess the independent value of different pretreatment variables on survival, in the presence of other variables, multivariate analysis was performed using the Cox proportional hazards model. Only variables with value 0.10 or less from the univariate analysis were entered into the Cox regression analysis. The significance level used in both univariate and multivariate analyses was P < 0.05, but in the post hoc subgroup analysis the significance level was moved from P = 0.05 to P = 0.01 due to risk of false positivity.

### Ethical clearance

The National Cancer Data Inspection Board and The Regional Committee for Research Ethics approved the study. The Regional Committee approved that written consent from the patients for their information to be stored in the hospital database and used for research was not needed because most of the material was more than 20 years old and most of the patients are now dead. The material was collected from our approved biobank for paraffin-embedded material and slides. All material was anonymously collected. The data were analyzed anonymously.

## Results

### Clinicopathological variables

The clinicopathological variables are summarized in Table 1. Median age was 59 (range, 0-91) years and 56% were female. The non-GIST STS comprised 249 tumors including pleomorphic sarcoma (n = 68), leiomyosarcoma (n = 67), liposarcoma (n = 34), malignant fibroblastic/myofibroblastic tumors (n = 20), rhabdomyosarcoma (n = 16), synovial sarcoma (n = 16), angiosarcoma (n = 13), malignant peripheral nerve sheath tumor (MPNST) (n = 11) and other types of sarcoma (n = 4). The tumors were localized in the extremities (n = 89), viscera (n = 58), trunk (n = 47), retroperitoneum (n = 37) and head/neck (n = 18). The treatment option of choice was surgery (n = 228), 120 patients received surgery alone, 55 patients received surgery and radiotherapy, 40 patients received surgery and chemotherapy and13 patients received surgery, radiotherapy and chemotherapy. Of the non-operated patients (inoperable, n = 11; advanced age/other serious disease, n = 5, STS diagnosis confirmed post mortem, n = 3; patient refusal, n = 2) seven received chemotherapy and/or radiotherapy. Fourteen patients did not obtain any treatment.

**Table 1:** Clinicopathological variables as predictors for disease-specific survival in 249 non-GIST STSs (univariate analyses, log-rank test).

Characteristic	Patients (n)	Patients (%)	Median survival (months)	5-Year survival (%)	P
**Age**					
≤ 60 years	133	53	59	50	0.065
> 60 years	116	47	30	40	
**Gender**					
Male	110	44	41	46	0.390
Female	139	56	45	45	
**Patient nationality**					
Norwegian	167	67	63	51	0.011
Russian	82	33	22	34	
**Histological entity**					
Pleomorphic sarcoma	68	27	29	40	0.102
Leiomyosarcoma	67	27	45	46	
Liposarcoma	34	14	NR	67	
MF/MFT	20	8	43	50	
Angiosarcoma	13	5	10	31	
Rhabdomyosarcoma	16	6	17	38	
MPNST	11	5	49	45	
Synovial sarcoma	16	6	31	29	
Other STSs	4	2	NR	18	
**Tumor localization**					
Extremities	89	36	100	53	0.348
Trunk	47	29	32	44	
Retroperitoneum	37	25	25	38	
Head/Neck	18	7	15	41	
Visceral	58	23	30	42	
**Tumor size**					
≤ 5 cm	74	30	127	57	0.027
5-10 cm	91	37	44	45	
> 10 cm	81	32	28	36	
Missing	3	1			
**Malignancy grade**					
1	61	25	NR	74	<0.001
2	98	39	41	45	
3	90	36	16	26	
**Tumor depth**					
Superficial	17	7	NR	93	<0.001
Deep	232	93	36	42	
**Metastasis at the time of diagnosis**					
No	206	83	76	53	<0.001
Yes	43	17	10	10	
**Surgery**					
Yes	228	92	59	50	<0.001
No	21	8	5	0	
**Resection margins**					
Free	178	71	127	66	<0.001
Not free/no surgery	71	29	10	18	
**Chemotherapy**					
No	191	77	52	47	0.424
Yes	58	23	29	40	
**Radiotherapy**					
No	176	71	48	46	0.590
Yes	73	29	38	43	

Abbreviations: non-GIST STS, non-gastro intestinal stromal tumor soft-tissue sarcoma; NR, not reached; MF/MFT, malignant fibroblastic/myofibroblastic tumors; MPNST, malignant peripheral nerve sheath tumor

### Interobserver variability

Interobserver scoring agreement was tested for all markers. The intraclass correlation coefficients were as follows: 0.89 for p-Akt Ser^473 ^(p < 0.001), 0.94 for p-Akt Thr^308 ^(p < 0.001), 0.91 for Akt2 (p < 0.001), 0.95 for Akt3 (p < 0.001), 0.88 for PI3-K (p < 0.001) and 0.89 for PTEN (p < 0.001).

### Expression pattern and correlations with clinicopathological variables

In the immunohistochemical analyses, we used antibodies against all Akt isoforms, including Akt phosphorylated at Ser^473 ^and at Thr^308^, non-phosphorylated Akt2 and total (both phosphorylated and non-phosphorylated) Akt3. Besides, we investigated expression of total PI3K and PTEN. The p-Akt Ser^473^, p-Akt Thr^308^, Akt2, Akt3, PI3K and PTEN showed expression in the cytoplasm or both in the cytoplasm and in the nuclei of tumor cells in the majority of cases, while pure nuclear staining was demonstrated in a smaller proportion of the tumors, varying from 7% of all immunohistochemically positive tumors for PTEN to 19% for p-Akt Thr^308 ^and Akt3.

Expression of p-Akt Ser^473 ^(r = 0.179, P = 0.005), p-Akt Thr^308 ^(r = 0.150, P = 0.019), Akt2 (r = 0.250, p < 0.001) and PI3K (r = 0.223, p < 0.001) correlated significantly positive with STS histological grade. PI3K and p-Akt Thr^308 ^positivity in STSs correlated with presence of metastasis at the time of diagnosis. Strong expression of p-Akt Thr^308 ^was observed in 69% of the metastasizing tumors, whereas only 41% of non-metastasizing STSs (r = 0.208, P = 0.001) were strongly positive for this marker. For PI3K, the metastasizing versus non-metastasizing characteristics comprised 78% and 53%, respectively (r = 0.188, P = 0.003). None of the investigated markers correlated significantly with age, gender, tumor location, depth, size or relapse rate.

### Univariate analyses

Data are presented in Table 1. Patient nationality (P = 0.011), tumor size (P = 0.027), malignancy grade (p < 0.001), tumor depth (p < 0.001), metastasis at time of diagnosis (p < 0.001), surgery (p < 0.001) and resection margins (p < 0.001) were all significant prognostic variables for DSS.

**Figure 2:**
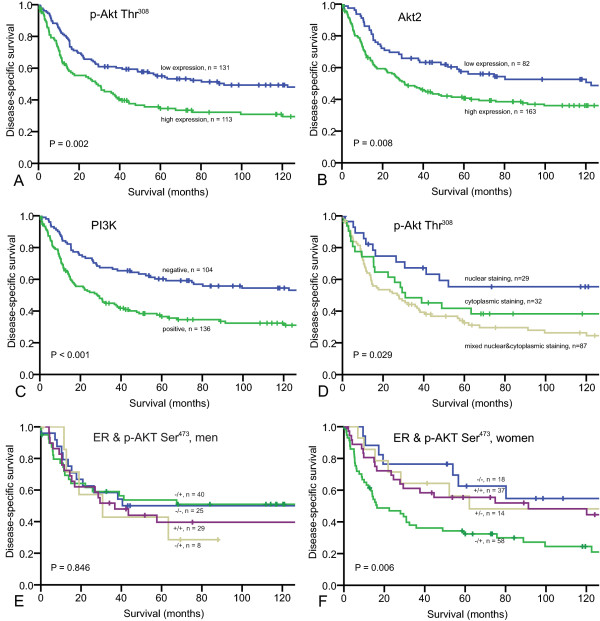
**Disease-specific survival curves for the investigated markers, their expression pattern and coexpression with steroid hormone receptors**. A, p-Akt Thr^308^; B, Akt2; C, PI3K; D, p-Akt Thr^308^, by cellular expression pattern; E, p-Akt Ser^473 ^in coexpression with ER, men; F, p-Akt Ser^473 ^in coexpression with ER, women. Abbreviations: p-Akt Thr^308^, Akt phosphorylated at threonin 308; PI3K, phosphoinositide 3-kinase; p-Akt Ser^473^, Akt phosphorylated at serin 473; ER, estrogen receptor.

The prognostic impact of the investigated molecular factors is shown in Table 2. Among these, p-Akt Thr^308 ^(P = 0.002), Akt2 (P = 0.008) and PI3K (p < 0.001) were significant indicators of shorter DSS, Figure 2, A-C.

**Table 2:** Tumor expression of markers belonging to PI3K/Akt signaling pathway and their prognostic impact on disease-specific survival in patients with non-GIST STSs (univariate analyses; log-rank test, n = 249), for all patients and separately for men and women.

Marker expression	Patients, n (%)			Median survival (months)			5-Year survival (%)			P		
	A	M	W	A	M	W	A	M	W	A	M	W
**p-Akt Thr^308^**												
Low	131(53)	59 (55)	72 (52)	91	NR	80	55	56	54	0.002	0.009	0.064
High	113 (45)	48 (44)	65 (47)	29	26	31	35	33	36			
Missing	5 (2)	3 (1)	2 (1)									
**p-Akt Ser^473^**												
Low	70 (28)	35 (32)	35 (25)	62	41	127	51	45	57	0.074	0.868	0.023
High	174 (70)	74 (67)	100 (72)	31	41	29	43	46	40			
Missing	5 (2)	1 (1)	4 (3)									
**Akt2**												
Low	82 (33)	41 (37)	41 (39)	123	NR	80	58	56	59	0.008	0.062	0.064
High	163 (65)	68 (62)	95 (68)	31	31	31	41	42	40			
Missing	4 (2)	1 (1)	3 (3)									
**Akt3**												
Low	177 (71)	81 (74)	96 (69)	62	63	57	51	51	50	0.067	0.207	0.197
High	60 (24)	22 (20)	38 (27)	31	27	38	35	33	36			
Missing	12 (5)	7 (6)	5 (4)									
**PI3K**												
Negative	104 (42)	44 (40)	60 (43)	NR	NR	127	60	57	63	<0.001	0.078	<0.001
Positive	136 (56)	61 (55)	75 (54)	29	37	23	37	41	33			
Missing	9 (4)	5 (5)	4 (3)									
**PTEN**												
Negative	88 (35)	37 (34)	51 (37)	80	NR	80	51	51	51	0.259	0.658	0.198
Positive	148 (59)	67 (61)	81 (58)	41	41	38	46	48	44			
Missing	13 (6)	5 (5)	7 (5)									

Abbreviations: Non-GIST STS, non-gastro intestinal stromal tumor soft-tissue sarcoma; A, all; M, men; W, women; NR, not reached

In order to find out whether subcellular location of proteins belonging to the Akt/PI3K signaling pathway has impact on survival, we performed a series of univariate analyses to compare the impact of their expression in nucleus, cytoplasm or both. Nuclear expression of p-Akt Thr^308 ^expression showed a significantly favorable prognosis (P = 0.029), compared to cytoplasmic and especially mixed cytoplasmic and nuclear expression, Figure 2, D. The other factors did not show any significant prognostic differences in the subcellular location.

Subgroup analysis based on clinical variables revealed that high expression of both p-Akt Thr^308 ^(P = 0.006) and Akt3 (P = 0.001) were adverse prognostic indicators for STSs located to extremities and for tumors larger than 5 cm in largest dimension (P = 0.001 for both markers). Interestingly, high expression of p-Akt Thr^308 ^was a negative prognostic factor particularly for men (P = 0.009 vs. P = 0.064 for women). In contrast, p-Akt Ser^473^, which appeared to be a negative prognosticator exclusively for female patients (P = 0.023 vs. P = 0.868 for men), Table 2.

### Multivariate Cox proportional hazards analyses

The results of the multivariate analysis are presented in Table 3. Advanced age of the patient (P = 0.038), deep site (P = 0.018), high malignancy grade (p < 0.001), metastasis at time of diagnosis (P = 0.010), lack of surgery (P = 0.031), non-free resection margins (p < 0.001), and PI3K expression by tumor cells (P = 0.042) were significant independent negative prognostic indicators of DSS.

**Table 3:** Results of the Cox regression analysis summarizing significant independent prognostic factors in the overall material.

Factor	Hazard Ratio	95% CI	P
**Age**			
≤60	1.0		
>60	1.5	1.0-2.1	0.038
			
**Tumor depth**			
Superficial	1.0		
Deep	11	1.5-79	0.018
			
**Malignancy grade**			<0.001*
1	1.0		
2	2.4	1.3-4.2	0.003
3	3.7	2.1-6.6	<0.001
			
**Metastasis at the time of diagnosis**			
No	1.0		
Yes	1.9	1.2-3.1	0.010
			
**Surgery**			
Yes	1.0		
No	2.2	1.1-4.4	0.031
			
**Resection-margins**			
Free	1.0		
Non-free	2.5	1.6-3.8	<0.001
			
**PI3K**			
Negative	1.0		
Positive	1.5	1.0-2.2	0.042

Abbreviations: PI3K, phosphoinositide-3-kinase.

* Overall significance as a prognostic factor

### Co-expression of activated Akt and PI3K with female steroid hormone receptors

The co-expression profiles of both types of activated Akt and PI3K with female steroid hormone receptors, in the group as a whole and stratified into gender were tested as shown in Table 4. The co-expression phenotypes PgR+/p-Akt Thr^308^+ among men (P = 0.023, HR = 2.4, 95% CI = 1.1-5.2), ER-/PI3K+ both in whole cohort (P = 0.005, HR = 2.0, 95% CI = 1.2-3.2) and among women (P = 0.014, HR = 2.4, 95% CI = 1.2-4.8), as well as PgR-/PI3K+ (P = 0.007, HR = 1.9, 95% CI = 1.2-3.0) and PgR+/PI3K+ (P = 0.014, HR = 1.9, 95% CI = 1.1-3.2) in the whole cohort of patients were significant independent negative prognostic factors. Interestingly, both steroid hormone receptors and Akt phosphorylation site seem to have opposite prognostic impact depending on the gender. This was further proved by the co-expression of these factors. Indeed, PgR-/p-Akt Ser^473^+ phenotype tended to have an unfavorable impact in women (P = 0.087) but was favorable in men (P = 0.010). Co-expression of ER and p-Akt Ser^473 ^showed similar results, with significantly adverse influence of -/+ profile on DSS among female patients (P = 0.006). There was no significant difference among the four possible profiles in men, but the -/+ curve demonstrated the best survival rate, Figure 2E and 2F.

**Table 4:** Co-expression of activated AKT and PI3K with ER and PGR and their prediction for DSS in patients with non-GIST STSs (univariate analyses; log-rank test, n = 249, only significant combinations are represented) and results of Cox regression analysis (multivariate analyses).

Univariate analyses						Multivariate analyses		
**Markers** **coexpression**	**Patients** **(n)**	**Patients** **(%)**	**Median survival** **(months)**	**5-Year survival** **(%)**	**P**	**Hazard ratio**	**95% CI**	**P**

**ER/p-Akt Thr^308 ^all**								
-/-	83	33	127	57	0.002			NS
-/+	58	23	18	29				
+/-	42	17	63	54				
+/+	47	19	45	46				
Missing	19	8						
**ER/p-Akt Thr^308 ^women**								
-/-	42	30	57	50	0.012			NS
-/+	35	25	16	27				
+/-	27	19	91	59				
+/+	25	19	120	53				
Missing	11	7						
**PgR/p-Akt Thr^308 ^all**								
-/-	101	41	127	59	0.014			NS
-/+	62	25	26	38				
+/-	26	10	54	46				
+/+	49	20	32	32				
Missing	11	4						
**PgR/p-Akt Thr^308 ^men**								
-/-	49	45	NR	64	0.003	1.0		0.099*
-/+	30	27	29	43		2.0	1.0-4.1	0.047
+/-	8	7	15	25		1.8	0.65-4.8	0.261
+/+	17	16	17	18		2.4	1.1-5.2	0.023
Missing	6	5						
**ER/p-Akt Ser^473 ^women**								
-/-	18	13	127	63	0.006			NS
-/+	58	41	16	32				
+/-	14	10	62	56				
+/+	37	27	91	55				
Missing	13	9						
**PgR/p-Akt Ser^473 ^men**								
-/-	29	27	NR	55	0.010	1.0		0.022*
-/+	51	47	NR	57		1.1	0.54-2.4	0.744
+/-	4	4	21	0		6.7	1.9-24	0.003
+/+	21	19	15	24		1.5	0.67-3.2	0.329
Missing	5	5						
**ER/PI3K all**								
-/-	65	27	127	60	0.002	1.0		0.032*
-/+	73	29	18	36		2.0	1.2-3.2	0.005
+/-	33	13	NR	63		1.1	0.56-2.1	0.816
+/+	56	22	37	43		1.4	0.83-2.4	0.200
Missing	22	9						
**ER/PI3K women**								
-/-	33	24	100	59	<0.001	1.0		0.036*
-/+	42	30	15	25		2.4	1.2-4.8	0.014
+/-	23	16	NR	70		0.9	0.36-2.0	0.715
+/+	29	21	29	46		1.5	0.72-3.0	0.290
Missing	13	9						
**PgR/PI3K all**								
-/-	76	31	NR	62	0.001	1.0		0.032*
-/+	86	35	29	43		1.9	1.2-3.0	0.007
+/-	26	10	100	58		1.3	0.69-2.5	0.397
+/+	46	18	31	27		1.9	1.1-3.2	0.014
Missing	15	6						
**PgR/PI3K men**								
-/-	36	34	NR	65	0.014			NS
-/+	44	40	63	51				
+/-	7	6	21	29				
+/+	15	14	17	20				
Missing	7	6						
**PgR/PI3K women**								
-/-	40	29	80	59	0.007			NS
-/+	42	30	17	36				
+/-	19	14	NR	68				
+/+	31	21	31	31				
Missing	8	6						

Abbreviations: ER, estrogen receptor; PgR, progesterone receptor; NR, not reached; NS, not significant;

* overall significance as a prognostic factor

## Discussion

In this large-scale retrospective study we have investigated the prognostic impact of a set of biomarkers belonging to the Akt-PI3K signaling pathway in non-GIST STS patients, both separately and in relation to gender. Further, we have also elucidated the coexpression of these markers and the female hormone receptors ER and PgR. These proteins participate in a diversity of processes in physiological and pathological conditions, especially in cancer development and progression [14]. p-Akt Thr^308^, Akt2 and PI3K showed significant unfavorable influence on survival of the whole cohort of patients in univariate analyses and, in addition, high expression of PI3K was a significant independent negative prognostic factor. p-Akt Thr^308 ^expression had a strong unfavorable impact among men, but was not significant in women. p-Akt Ser^473 ^expression had strong adverse impact in women but was not significant in men or in the whole cohort. PgR-/p-Akt Ser^473^+ phenotype showed less favorable impact in women, but was the most favorable one in men. To our knowledge, this is the first prognostic evaluation of these biomarkers in non-GIST STSs.

Akt, aka protein kinase B, is a serine/threonine protein kinase. Currently, three mammalian isoforms (Akt1/PKBα, Akt2/PKBβ, and Akt3/PKBγ) have been identified. They are encoded by different genes and have different tissue distribution [29].

In a healthy organism, Akt1 is a key signaling protein in the cellular pathways that result in skeletal muscle hypertrophy, and general tissue growth [30]. Akt can be phosphorylated by its two activating kinases, phosphoinositide dependent kinase 1 (PDK1) - at threonine ^308^, and mammalian target of rapamycin complex 2 (mTORC2), previously putatively named PDK2, - at serine ^473^. Both mTORC2 and PDK1 are products of the PI3K pathway. Activated Akt can activate or deactivate its multiple substrates, including mammalian target of rapamycin (mTOR), bcl-2 family member BAD, transcription factor forkhead homolog 1 in rhabdomyosarcoma (FKHR), Mdm2 protein, glycogen synthase kinase 3 (GSK3) and many others, via its kinase activity [31,32].

Akt1 is involved in cellular survival pathways by inhibiting apoptotic processes. Since it thereby promotes cell survival, Akt1 has been regarded as a major factor in many types of cancer [15-17]. The majority of studies agree that high expression of Akt by tumor cells indicates a poor prognosis [19-21]. However, in a recent study by Baba et al., phosphorylated Akt expression was reported to have a favorable impact on DSS in 717 colorectal cancer patients [22]. Similar results were obtained by Mori et al. in a study devoted to Akt expression in endometrial carcinoma [23]. This discrepancy can probably be explained by the site of Akt phosphorylation. Both studies utilized antibodies against p-Akt Ser^473^, while the articles describing negative influence of Akt are based on p-Akt Thr^308 ^expression [20,33,34]. Al-Saad et al. [33] has recently compared the prognostic impact of Akt phosphorylated on both sites and demonstrated that expression of p-Akt Thr^308^, unlike p-Akt Ser^473^, negatively influenced prognosis in patients with non-small cell lung cancer.

For the whole cohort we also found that p-Akt Thr^308 ^expression was associated with a shorter STS survival in univariate analyses, while p-Akt Ser^473 ^expression had no significant value. However, calculated separately for each gender, high expression of p-Akt Thr^308 ^was a negative prognostic factor particularly for men, in contrast to p-Akt Ser^473^, which appeared to be a negative prognosticator exclusively for female patients. This prompted us to further investigate this phenomenon by studying of co-expression profiles of both types of activated Akt with female steroid hormone receptors. In our previous works we have shown that ER and PgR expression possess variable prognostic significance depending of gender both *per se *[25] and in co-expression with TGF-β and fascin [26]. ERβ was shown to activate PI3K/Akt signalling pathway [35]. Tsai et al. demonstrated an activation of Akt by estrogen in ER negative breast cancer cell culture [36]. In the present study, the prognostic diversity of these factors in men and women was enhanced in the co-expression profiles: male patients with STSs expressing simultaneously p-Akt Thr^308 ^and PgR had statistically significant minimal survival rate. For women, the ER-/p-Akt Ser^473^+ expression profile was the most unfavorable phenotype.

Taking into consideration a possible distortion of the results by gender-related sarcomas (i.e. leiomyosarcoma in uterus) we have attempted to exclude these sarcomas and recalculate all analyses. There were no considerable differences in the results by exclusion of gender-related sarcomas comparing to those obtained for whole cohort (data not shown).

Akt2 is an important molecule in the insulin signaling pathway, but in Akt1 deficient mice it is also proved to substitute, at least partly, the role of Akt1 in growth and proliferation [37]. We found Akt2 expression to be associated with significantly shorter DSS in univariate analysis. This might be explained by the extra-endocrine function of Akt2. The role of Akt3 is less clear, it appears to be predominantly expressed in the central nervous system [29]. In this study, we failed to demonstrate any association of Akt3 with the survival of STS patients.

PI3K is, via PDK1 and mTORC2 dependent activation, an upstream regulator of all Akt isoforms, and plays an important role in the PI3K/Akt pathway. Its high expression has been implicated as an adverse prognostic factor in many types of cancer [38-40]. In STS, we observed that PI3K expression was an independent significant indicator of shorter DSS. Not surprisingly, the co-expression of PI3K with both ER and PgR showed multiple independent negative impacts on survival in STS patients with the phenotypes ER-/PI3K+ in women and PgR+/PI3K+ in men being the least favorable.

The tumor suppressor gene PTEN negatively regulates the PI3K/Akt signaling pathway. It is a proapoptotic and antineoplastic factor and shown to be a favorable prognosticator in cancer patients [41]. In our study, we failed to find any statistical difference in survival between patients having PTEN-positive and PTEN-negative STSs.

In our material, all investigated factors showed three distinct patterns of expression; nuclear, cytoplasmic and combined nuclear and cytoplasmic. Little is known about the prognostic value of such subcellular stratification. Le Page et al. reported that nuclear Akt-1 and Akt-2 expression were significantly correlated with favorable outcome in 63 prostate cancer patients, while cytoplasmic Akt-1 expression was correlated with a higher risk of postoperative prostate-specific antigen (PSA) recurrence and shorter PSA recurrence interval [42]. In the present study, we were able to find such dependence only for nuclear p-Akt Thr^308 ^expression, which proved to be prognostically favorable compared to cytoplasmic and especially mixed cytoplasmic and nuclear location.

## Conclusion

We have characterized the occurrence and distribution of several proteins belonging to PI3K/Akt signaling pathway in STS patients with respect to tumor aggressiveness and DSS. Our findings are largely in agreement with the results of a number of studies that have investigated the roles of these markers in other, especially epithelial, tumors. Nevertheless, the diverse prognostic values depending on the site of Akt phosphorylation and on the co-expression with female steroid hormones have not been described earlier.

Our findings may help to identify subgroups of patients with aggressive tumors requiring adjuvant therapy which, due to relationship of the PI3K/Akt pathway components with female steroid hormone receptor proteins, could include specific endocrine treatment. Moreover, since the investigated biomarkers belong to the family of serine-threonine kinases, which are comprehended as "drugable"[18], they may represent molecular targets for personalized, small-molecule targeted therapy. This currently is a hotspot of oncological research, and it was shown effect of such Akt targeted agents on several subtypes of sarcomas in vitro [43,44].

## Competing interests

The authors declare that they have no competing interests.

## Authors' contributions

AV, SWS, TKK, TD, RMB and LTB participated in the design of the study. AV ES and TKK collected clinical information. AV and SWS reviewed all the histological diagnoses, histological grading, selected and marked the slides for TMA construction. AV, TKK and SWS performed the experiments. AV, TKK, SWS, TD, RMB and LTB performed the statistical analysis. AV, TK, SWS, TD, ES and LTB contributed reagents/materials/analysis tools. AV, TD, ES, RMB and LTB drafted the manuscript. All authors read and approved the final manuscript.
